# A novel mouse model of heart failure with preserved ejection fraction after chronic kidney disease induced by retinol through JAK/STAT pathway

**DOI:** 10.7150/ijbs.83432

**Published:** 2023-07-16

**Authors:** Bowen Liu, Adilan Shalamu, Zhiqiang Pei, Liwei Liu, Zilun Wei, Yanan Qu, Shuai Song, Wei Luo, Zhen Dong, Xinyu Weng, Junbo Ge

**Affiliations:** 1 Department of Cardiology, Zhongshan Hospital, Shanghai Institute of Cardiovascular Diseases, Fudan University, Shanghai, 200000, China.; 2 National Clinical Research for Interventional Medicine, Shanghai, 200000, China.; 3 Key Laboratory of Viral Heart Diseases, National Health Commission, Chinese Academy of Medical Sciences, Shanghai, 200000, China.; 4 Institutes of Biomedical Sciences, Fudan University, Shanghai, 200000, China.; 5 Department of Cardiology, Taiyuan Central Hospital of Shanxi Medical University, Shanxi, 030000, China.

**Keywords:** heart failure with preserved ejection fraction, chronic kidney disease, retinol, animal model, JAK/STAT

## Abstract

Heart failure is the leading cardiovascular comorbidity in chronic kidney disease (CKD) patients. Among the types of heart failure according to ejection fraction, heart failure with preserved ejection fraction (HFpEF) is the most common type of heart failure in CKD patients. However, the specific animal model of HFpEF afer CKD is currently missing. In this study, we determined the heart failure characteristics and dynamic progression in CKD mice. Based on these features, we established the practical HFpEF after CKD mouse model using 5/6 subtotal nephrectomy and retinol administration. Active apoptosis, impaired calcium handling, an imbalance between eNOS and oxidative stress and engaged endoplasmic reticulum stress were observed in our model. RNSseq revealed distinct gene expression patterns between HFpEF after CKD and metabolic induced-HFpEF. Furthermore, we revealed the potential mechanism of the pro-HFpEF effect of retinol. Serum accumulation of retinol in CKD prompts myocardial hypertrophy and fibrosis by activating JAK2 and phosphorylating STAT5. Finally, using small molecule inhibitor AC-4-130, we found STAT5 phosphorylation inhibitor may be a potential intervention target for HFpEF after CKD. In conclusion, we provide a novel animal model and a potential drug target for HFpEF intervention in CKD.

## Introduction

As a clinical common disease affecting multiple organs throughout the body, chronic kidney disease (CKD) impacts 9.1% global population causing severe burden and various complications from cognitive impairment to peripheral arterial stiffness 1-4. Heart failure is the leading cardiovascular comorbidity in CKD patients with a high incidence of 18/1000 person-years and a 10- to 100-fold increase of cardiovascular death risk 5, 6. Among the types of heart failure according to ejection fraction, heart failure with preserved ejection fraction (HFpEF) is the most common type of heart failure in CKD patients manifesting as diastolic dysfunction and preserved ejection fraction 7, 8. However, HFpEF is a multifactorial syndrome of various etiologies, initiating and developing from multiple factors during patients lifetime 9. Due to the complexity of etiologies, the heterogeneity of subtypes and the ambiguity of pathogenesis, the mortality for HFpEF remains high and the targeted drug is rare except the newly reported drug sodium-glucose cotransporter 2 inhibitor 10, 11. Therefore, determining the triggering factors and molecular processes of HFpEF in CKD patients is the key for intervention. Because of the limited access to human samples, a representative and convenient animal model of HFpEF after CKD is the cornerstone for pathogenesis identification and drug discovery.

Considering the different factors driving HFpEF, the practicable animal models of HFpEF can also be classified into 3 subtypes according to the accompanying complications, such as metabolic stress induced db/db mouse model and 2-hit mouse model, hypertension induced AIU mouse model and aortic coarctation-induced AC feline model 12-16. As a crucial risk factor and indispensable type of HFpEF, neither one of HFpEF model covers CKD and also none model could be used to represent and to simulate the actual pathophysiological state of tens of millions HFpEF after CKD patients worldwide 17-18. The difference between HFpEF after CKD and other types of HFpEF in molecular phenotype remains unknown, and the unique process of HFpEF after CKD is still unclear due to the lack of specific animal model.

In this study, we established the practical HFpEF after CKD mouse model by using subtotal nephrectomy and retinol administration. We also determined the optimum observation and operation window for HFpEF after CKD in our mouse model. Cardiomyocyte hypertrophy, interstitial fibrosis, apoptosis, impaired calcium handling, endoplasmic reticulum stress and macrophage infiltration were observed in HFpEF after CKD mouse model. The differences of gene expression patterns between HFpEF after CKD and metabolic stress-induced HFpEF were revealed. And we demonstrated retinol may prompt HFpEF progression in CKD through Stra6-JAK2-STAT5 signals and restraining phosphorylation of STAT5 may attenuated HFpEF development in CKD by using STAT5 phosphorylation inhibitor AC-4-130.

## Materials and methods

### Animals and treatments

Male C57BL/6J mice aged 5 weeks (weight 16-21g) were purchased from Gem Pharma Tech LLC (Nanjing, Jiangsu, China). All mice were fed in a 12/12-h light/dark cycle room at 24 ± 1 °C and 60% ± 10% humidity. Animals are free to access water and normal diet containing 4000 IU of vitamin A/kg or a vitamin A-free AIN-93G diet (Oriental Yeast Co., Ltd). Before surgery, mice were synchronized to new environment for 2 weeks. At the age of 7 weeks, CKD mice were subjected to two step 5/6 nephrectomy(5/6Nx). First, two-third of the left kidney were removed by cutting upper and lower poles and gelatin sponge hemostasis. After seven days recovery, the right kidney was ligated and removed entirely. After the operation, mice housed and given 0.5ml 300mg/kg vitamin A water solution (Amresco Co., Ltd) every day for 6 weeks to establish CKD-HFpEF. Sham mice were subjected to laparotomy on the same days as the 5/6Nx surgeries. To establish the suitable mouse model of HFpEF after CKD, typical HFpEF inducing methods such as 1% NaCl administration referring to AIU HFpEF mouse model, L- NG- nitro arginine methyl ester (L-NAME) or high-fat diet from 2-hit HFpEF mouse model were administrated in 5/6 nephrectomy mice. To downregulate Stra6, anti-Stra6 siRNA or control siRNA were injected into mice (40 μg/week; Life Technologies) encapsulated in HVJ-E (Cosmo Bio) through tail vein every week for 6 weeks during vitamin A administration. The siRNA oligonucleotide sequences were as follows: anti-Stra6 siRNA sense 5′-GCUGCUGUCUUUGUGGUCCUCUUCA-3′ and antisense 5′-UGAAGAGGACCACAAAGACAGCAGC-3′; control siRNA sense, 5′-GCUGUCUUUGUGUUGCCCUUCGUCA-3′ and antisense 5′-UGACGAAGGGCAACACAAAGACAGC-3′. Mice were also injected with either STAT5 SH2 domain small molecule inhibitor AC-4-130 (25mg/kg) or vehicle (10% DMSO, 5% Cremophore in PBS). AC-4-130 administration via intraperitoneal injection was well tolerated with no toxicity. To evaluate the effect of AC-4-130 in CKD, mice were injected with either AC-4-130 (25mg/kg) or vehicle intraperitoneally in 8 weeks after first stage surgery of CKD for two weeks. We collected the blood and kidney tissues after 4 weeks of CKD surgery completion when mice were 13 weeks. Lepr^db/db^ mice (db/db mice) were purchased from Gem Pharma Tech LLC (Nanjing, Jiangsu, China) and raised for 20 weeks to develop HFpEF. All experimental procedures were conducted in accordance with the Guide for the Care and Use of Laboratory Animals published by the US National Institutes of Health (NIH publication no. 85-23, revised 1996), the Guide for the Care and Use of Laboratory Animals (National Research Council Publication, 8th Edition, 2011) and the Chinese Regulations for the Administration of Affairs Concerning Experimental Animals. The animal experiments were approved by the Animal Ethics Committee at Zhongshan Hospital, Fudan University (Shanghai, China). All the animal procedures were performed in the Department of Experimental Animal Research Centre, Zhongshan Hospital, Fudan University (Shanghai, China).

### Population and blood collection

The CKD patients (n=12) and HFpEF after CKD (CKD-HFpEF) patients (n=12) were enrolled in Zhongshan Hospital, Fudan University from April 2019 to December 2022. The inclusion criteria for CKD-HFpEF patients were as follows: (1) provision of a signed informed consent form, (2) age of ≥ 18, (3) diagnosed HFpEF based on current guideline definitions of HFpEF 19, 20 to include the following: signs and symptoms of clinical HF using Framingham criteria for HF 21, LV ejection fraction ≥50% by echocardiography within the previous 12 months, and at least 2 of the following: ① structural heart disease (increased LV wall thickness or left atrial diameter) or diastolic dysfunction on echocardiography 22; ② NT-proBNP (N-terminal pro-B type natriuretic peptide) ≥125 pg/mL; or ③ hemodynamic evidence of elevated left-sided filling pressures (pulmonary artery wedge pressure ≥15 mm Hg at baseline or ≥25 mm Hg with exercise), (4) diagnosed chronic kidney disease at least 1 year before the clinical HF symptoms presentation or HFpEF determination. Chronic kidney disease is defined as an eGFR between 25 and 75 ml/min/1.75 m^2^ and a urinary albumin-to-creatinine ratio (UACR) between 200 and 500 mg/g. The estimated glomerular filtration rate (eGFR) was calculated by the Modification of Diet in Renal Disease formula 23. The exclusion criteria for CKD-HFpEF patients were as follows: (1) history of LV ejection fraction <40% or midrange ejection fraction (40% to 50%), (2) hypertrophic cardiomyopathy, (3) restrictive cardiomyopathy, (4) infiltrative cardiomyopathy (including cardiac amyloidosis), (5) congenital heart disease or constrictive pericarditis, (6) greater than moderate valvular disease, (7) isolated pulmonary arterial hypertension, (8) heart transplantation, (9) diabetes, (10) lupus nephritis, lupus nephritis, (11) lupus nephritis, (12) anti-neutrophil cytoplasmic antibody-associated vasculitis. The inclusion criteria for CKD patients were as follows: (1) provision of a signed informed consent form, (2) age of ≥ 18, (3) diagnosed chronic kidney disease. The exclusion criteria for CKD patients were as follows: (1) heart failure, (2) hypertrophic cardiomyopathy, (3) restrictive cardiomyopathy, (4) infiltrative cardiomyopathy (including cardiac amyloidosis), (5) congenital heart disease or constrictive pericarditis, (6) greater than moderate valvular disease, (7) isolated pulmonary arterial hypertension, (8) heart transplantation, (9) diabetes, (10) lupus nephritis, lupus nephritis, (11) lupus nephritis, (12) anti-neutrophil cytoplasmic antibody-associated vasculitis. The baseline variables of the enrolled patients are shown in Table S1. The study protocol strictly complied with the ethical guidelines of the 1975 Declaration of Helsinki and was approved by the Zhongshan Hospital, Fudan University Ethics Committee. All participated patients were fully informed and signed the informed consent form before enrolment. 3 ml peripheral blood was collected by BD K2 EDTA Vacutainer and BD Serum Vacutainer (BD Co., Ltd), respectively and stored in 4°C refrigerators temporarily. All blood samples were centrifugated in 3500rpm, 4°C for 5 minutes within 6 hours after collection. Plasma and serum were separated and stored in -80°C refrigerators.

### Histochemical staining

Hearts, livers and skins were immersed in 4% paraformaldehyde in phosphate-buffered saline (PBS) for 12 h after removal. The processed tissues were stained with Hematoxylin Eosin (HE) and Masson's staining, respectively. For HE staining, tissues were soaked in 10% neutral buffered formalin (NBF) for 24 hours and embedded in paraffin, and cut into 4mm -thick slices for microscope observation. As for Masson staining, tissues were stained by Masson's reagent, rinsed by 15% or 30% sucrose and snap-frozen. Collagen volume fraction was calculated by dividing the blue-stained fibrotic area by the total area using Image J.

### Immunofluorescence histochemical staining

Frozen slices of heart were used for immunofluorescence histochemical staining. Cryostat cardiac sections fixed with 4% paraformaldehyde were blocked in solution containing 10% FBS and 0.1% Triton X-100 for 1 h at 4 °C, followed by incubation with antibodies against WGA (GDP1020, Servicebio), GRP78 (ab21685, Abcam), eNOS (ab300071, Abcam), Serca2(ab150435, Abcam) or F4/80 (ab6640, Abcam) at 4 °C for 24 h. After washing and incubating with a fluorescent secondary antibody (Alexa 488 or Alexa 546, Jackson ImmunoResearch, Laboratories, West Grove, PA) at 4 °C for 2 h, the slices were mounted using Vectashield hard-set mounting medium with 4′,6-diamidino-2-phenylindole (Vector Laboratories, Burlingame, CA, USA).

### TdT-mediated dUTP nick end labeling (TUNEL) assay

Paraffin sections of heart tissue were cut into 4mm-thick slices. Then, slices were washed with xylene two times for 5 min each and immersed in a graded series of ethanols. After that, proteinase K working fluid was added for 15-30 min at 21-37 °C. After two washes with PBS, the TUNEL-converter-POD solution reaction mixture was added and reacted with 3,3' diaminobenzidine (DAB) for colour development.

### Echocardiography

We performed transthoracic ultrasound cardiography on mice under lightly anesthesia. Two-dimensional targeted M-mode tracings were recorded using a diagnostic ultrasound system (SSA-700A, Toshiba) equipped with a 15 MHz transducer. The LVEF was calculated using Pombo's method. The %FS was calculated as follows: LVFS =[(LVIDd - LVIDs)/LVIDd] × 100.

### Blood pressure measurement

We used the non-invasive methods to measure the blood pressure of conscious mice by a computerized tail-cuff system (BP-2000, Visitech Systems, Apex, NC). First, mice were trained for 3 consecutive days to adapt to the procedure. Then, heart rate and blood pressure were record. For each mouse, 10 measurements were obtained and the mean values were calculated.

### Quantification of cytokines and biochemical indices

Serum concentrations of creatinine, urea nitrogen, angiotensin II, aldosterone, BNP, ALT, AST, KIM-1, NGAL, RBP4 were detected by using Creatinine Kit (BC4910, Solarbio), Blood Urea Nitrogen Kit (BC1535, Solarbio), Angiotensin II ELISA kit (ml002228, Mlbio), Aldosterone ELISA kit (ml037530, Mlbio), Brain Natriuretic Peptide EIA Kit (ml037594, Mlbio), ALT Kit (BC1555, Solarbio), AST Kit (BC1565, Solarbio), Mouse KIM-1 ELISA Kit (BC4910, Solarbio), Mouse NGAL ELISA Kit (ml002075, Mlbio), Mouse RBP4 ELISA Kit (ARG81756, Arigo). Plasma concentrations of IL-1β, IL-6 and TNF-α were measured by Mouse IL-1β ELISA Kit (ml301814, Mlbio), Mouse IL-6 ELISA Kit (ab222503, Abcam), Mouse TNF-α ELISA Kit (ab208348, Abcam). Serum levels of retinol and retinoic acid were measured by liquid chromatography/mass spectrometry analysis following the previous methods 24, 25.

### Exercise testing

Exercise capacity test was conducted in a 6-lane rodent treadmill system (47303, Ugo Basile). First, mice were put to run at low speed (1m/min) for 10 minutes to acclimatization. Then, exercise testing was performed by gradually elevating running speed (increasing 1m/min every 2 minutes). After mice left the lane and remained on a pad for 5s, maximal speed and running distance were record. Exercise capacity was demonstrated as workload, calculated as running distance multiplied by body weight.

### Measurement of pulmonary water content

After removing the lungs completely, we used bibulous paper to wipe off the blood and water on surface and record the weight of lung as lung wet weight. Then we wrap the lungs in tin foil and dry it in a thermotank at 72°C for 72 hours. After that, we weighed the dried lung and record it as lung dry weight. pulmonary water content was calculated by dividing lung dry weight by lung wet weight.

### Lipid Peroxidation (MDA) Assay

We used the Micro Malondialdehyde (MDA) Assay Kit (BC0025, Solarbio) to evaluate the level of lipid oxidation. After weighing 0.1g heart tissue, we add 1mL extracting solution and homogenize it in ice bath. Then, we centrifuge it at 8000g 4°C for 10min and take the supernatant. After adding the working solution to the supernatant, we can calculate the MDA concentration by a microplate reader.

### Western blotting

After adding cold RIPA lysis buffer (Beyotime, Shanghai, China) to heart tissues, we homogenized the tissues in ice bath for 3 min and then centrifuged at 16300g, 4°C for 20 min. We collect the supernatant and evaluate the protein concentration by using Enhanced BCA Protein Assay Kit (Beyotime, Shanghai, China). Equal amounts of protein were separated by SDS-PAGE and electrophoretically transferred to PVDF membranes. Then we blocked the membranes in Blocking buffer for 2h and then incubated it with primary antibodies overnight at 4°C. Antibodies are described below: anti-Bax (Cell Signaling Technology, #14796, 1:1000), anti-Bcl-2 (Abcam, ab196495, 1:10000), anti-Caspase 3 (Cell Signaling Technology, #9662, 1:1000), anti-Serca2 (Abcam, ab150435, 1:2000), anti-GRP78 (Abcam, ab 21685, 1:2000), anti-CHOP (Cell Signaling Technology, #2895, 1:1000), anti-JAK2 (ab108596, Abcam), anti-STAT5 (ab230670, Abcam), anti-p-STAT5 (ab32364, Abcam). Expression levels were normalized by probing the same blots with an anti-GAPDH antibody (Cell Signaling Technology, #5174).

### RNA sequencing and data analysis

The heart tissue of Sham group (n=6), CKD-HFpEF group (n=6) and db/db group (n=6) were sent for RNA sequencing. The RNA was sequenced by next-generation RNA sequencing using Illumina HiSeqTM 2000 in Novogene (Tianjin, China). Differentially expressed genes were screened using the following criteria: (1) fold change >2 for upregulation or fold change<0.5 for downregulation, (2) P value <0.05. Principal component analysis and venn diagram was used to demonstrate the similarity and difference between CKD-HFpEF group and db/db group. GO and KEGG analysis were applied to predict the underlying mechanism and pathways. the protein-protein interaction network was also drawn to predict the possible relationship among differentially expressed molecules.

### RNA collection and Quantitative RT-PCR

RNA was extracted with TRIzol™ Reagent (#15,596,026, Invitrogen). The concentration and purity of RNA were assessed with NanoDrop 2000 spectrophotometer (Thermo Fisher Scientific). Samples with 1000 ng or higher and an A260/A280 ratio of 1.8-2.0 were considered qualified for use. Qualified RNA was stored at -80°C for further analysis. cDNA was reverse transcribed using PrimeScrip Reverse Transcription Master mix (# RR036A, TaKaRa). A total of 20 μL reaction system was used, including DNA template 1.6 μL and SYBR 10 μL, and primers 0.4 μL and ddH2O 7.6 μL. Primers used in this study were as follows: JAK2 forward primer: 5′-GGAATGGCCTGCCTTACAATG-3′, JAK2 reverse primer: 5′-TGGCTCTATCTGCTTCACAGAAT-3′, STAT5 forward primer: 5′-CAGCCGTGGGATGCTATTGA-3′, STAT5 reverse primer: 5′-GGGACAGCGGTCATACGTG-3′. The quantitative PCR was performed in a CFX Connect Real-Time system (Bio-Rad, USA) and the PCR reaction cycles were set as follows: 30 s at 95 °C, then 5 s at 95 °C and 30 s at 60 °C for 40 cycles. The relative expression levels were normalized to those of the reference gene GAPDH and calculated using the 2^-▲▲CT^ method. All reactions were repeated in triplicate.

### Statistical analysis

Continuous variables are presented as means±SEs or medians (with 25th and 75th percentiles). Student's t test was used to determine the differences between two groups of Gaussian distributed variables. Among multiple groups of Gaussian distributed variables, ANOVA was used to tell the differences. While variables that were not Gaussian distributed, Wilcoxon rank sum test and Kruskal-Wallis test were used for differences between two groups and more than two groups of variables, respectively. Categorical variables are reported as percentages and were analysed by either the chi-square test or Fisher's exact test. Pearson correlation analysis and Spearman correlation analysis were performed to analyse Gaussian and non-normally distributed variables, respectively. Receiver operating characteristic curves were used to establish cut-off values for HFpEF after CKD predictions. All P values are 2-sided, and P values of less than 0.05 were considered statistically significant. All statistical analyses were carried out by using SPSS 16.0 and R 3.3.0 software.

## Results

### CKD mice can develop heart failure spontaneously followed by LVEF and LVFS decline

**Figure 1:**
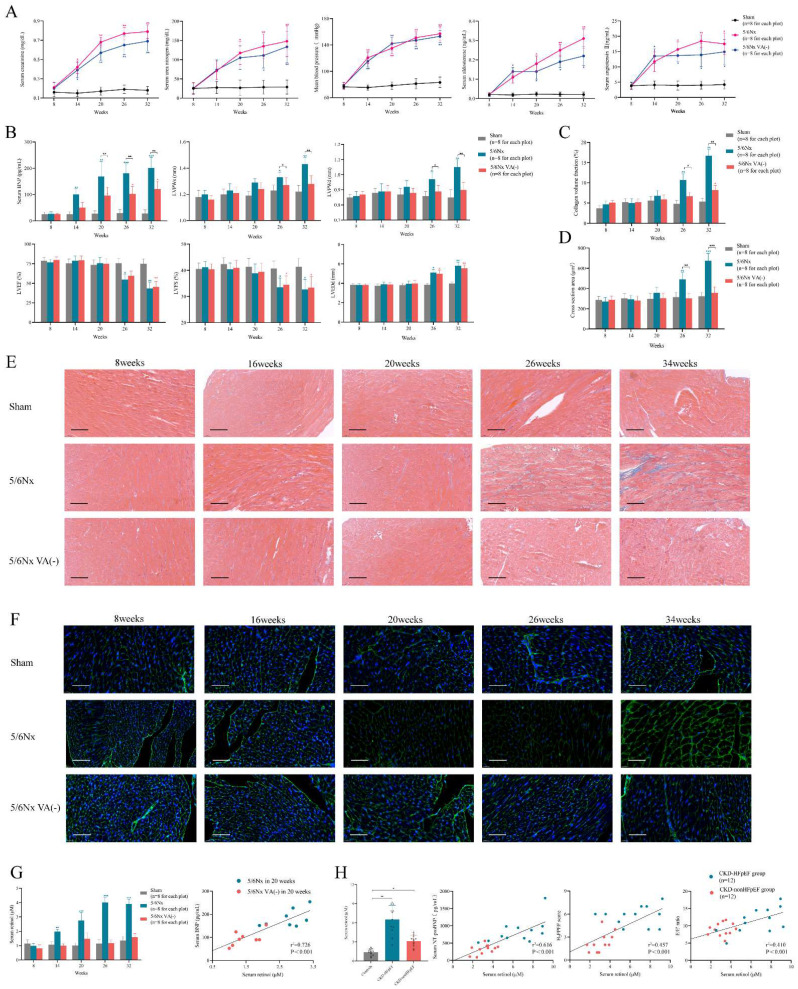
Evaluation of the spontaneous heart failure CKD mouse model develops without induction. **(A)** Serum concentrations of creatinine, urea nitrogen, aldosterone, angiotensin Ⅱ and mean blood pressure in 5/6Nx mice, 5/6Nx mice fed with vitamin A free diet and sham-operated mice from 8 weeks to 32 weeks. **(B)** Serum BNP concentration, LVPWs, LVPWd, LVEF, LVFS and LVEDd in 5/6Nx mice, 5/6Nx mice fed with vitamin A free diet and sham-operated mice from 8 weeks to 32 weeks. **(C)** Collagen volume fraction calculated from Masson staining of the heart tissues from 5/6Nx mice, 5/6Nx mice fed with vitamin A free diet and sham-operated mice from 8 weeks to 32 weeks. **(D)** Cross section area calculated from WGA staining of the heart tissues from 5/6Nx mice, 5/6Nx mice fed with vitamin A free diet and sham-operated mice from 8 weeks to 32 weeks. **(E)** Masson staining in the cardiac ventricle of 5/6Nx mice, 5/6Nx mice fed with vitamin A free diet and sham-operated mice from 8 weeks to 32 weeks. **(F)** WGA staining in the cardiac ventricle of 5/6Nx mice, 5/6Nx mice fed with vitamin A free diet and sham-operated mice from 8 weeks to 32 weeks. **(G)** The left panel shows serum retinol concentration in 5/6Nx mice, 5/6Nx mice fed with vitamin A free diet and sham-operated mice. The right panel shows the relationship between serum retinol and BNP in 5/6Nx mice and 5/6Nx mice fed with vitamin A free diet at week 20. Each plot shows a value obtained using an individual mouse serum. **(H)** Relationship serum retinol level to serum NT-proBNP concentration, H_2_FPEF score or E/E' ratio in CKD patients. Each plot shows a value obtained using an individual human serum. Left panel shows the serum retinol concentration in CKD patients and HFpEF after CKD patients. Data are shown as mean ± SD (*p < 0.05, **p < 0.01, ***p < 0.001).

First, we evaluated the classic chronic kidney disease (CKD) mouse model in a long observation period, in order to determine whether CKD model can develop into heart failure with preserved ejection fraction (HFpEF) over time spontaneously without drug induction. The 5/6 subtotal nephrectomy (5/6Nx) model is a classical CKD mice model which is widely used to mimic human CKD in mice. 5/6 subtotal nephrectomy can induce the progressive renal failure involving glomerulosclerosis and tubulointerstitial fibrosis, which present as persistent serum creatinine elevation for more than 4 weeks 26, 27. After 5/6Nx surgery completion in 8 weeks, we observed serum creatinine and urea nitrogen climbing quickly from 2-fold increase in 14 weeks to over 10-fold increase in 32 weeks, which fulfilled the criteria of CKD. Renin-angiotensin-aldosterone system (RAAS) was upregulated manifested as serum aldosterone and angiotensin Ⅱ rise. Accompanying with the renal function deterioration, mean blood pressure was also elevated from 78mmHg before surgery to 128mmHg 24 weeks after surgery (Fig. 1A).

Not like renal function and blood pressure, cardiac function decline and myocardial hypertrophy were appeared andante. Though brain natriuretic peptide (BNP) level was progressing right after surgery, LVPWs and LVPWd were significantly increased 18 weeks after surgery (Fig. 1B). Masson and WGA staining also demonstrated evident interstitial fibrosis and myocardiocyte hypertrophy in the meantime (Fig. 1C-F). But the synchronous decline of LVEF and LVFS clarified it was heart failure with reduced ejection fraction (HFrEF) not HFpEF which CKD model developed into without intervention, which was obviously inconsistent with heart failure presentation in clinical CKD patients (Fig. 1B). Therefore, the distinction of cardiac function and long induction time made it was not appropriate to use CKD mouse model directly to represent and simulate HFpEF after CKD patients.

### Elevated retinol in CKD promotes myocardial hypertrophy and fibrosis

To establish the suitable mouse model of HFpEF after CKD, we used the typical HFpEF inducing methods to induce HFpEF after CKD such as 1% NaCl administration referring to AIU HFpEF mouse model, L- NG- nitro arginine methyl ester (L-NAME) or high-fat diet from 2-hit HFpEF mouse model. However, 1% NaCl or L- NG- nitro arginine methyl ester (L-NAME) injection caused high death rate and high-fat diet could not induce HFpEF in CKD. Therefore, we reviewed the articles of heart failure in CKD to find an appropriate way to induce HFpEF after CKD. Recent research reported retinol could prompt heart failure occurrence in CKD by accelerating inflammation and fibrosis through Clock/Arntl/GPR68 signal 28. We verified serum retinol level was increased by 4 times after 5/6Nx and was positively correlated with BNP level in CKD mouse model (Fig. 1G). We also enrolled 36 patients to perform a case-control study with 12 HFpEF after CKD cases, 12 CKD without heart failure cases and another 12 controls. Clinical characteristics were collected, and the baseline data was displayed in Table S1. Retinol was significantly higher in HFpEF after CKD patients than CKD without heart failure patients or controls. And the retinol level was positively correlated with NT-proBNP levels, H_2_FPEF score and E/E' ratio respectively in HFpEF after CKD patients and CKD without heart failure patients (Fig. 1H). Vitamin A is derived from the diet, mostly as retinol esters. As retinol is the main circulating retinoid, dietary deficiency of vitamin A reduces serum retinol levels 29. Feeding 5/6Nx mice with a vitamin A-free diet reduced retinol level to the control level (Fig. 1G). Vitamin A-free diet did not attenuate the progress of renal decline, hypertension and RAAS activation after 5/6Nx (Fig. 1A). But it effectively decreased LVPWs, LVPWd, myocardiocyte cross section area and collagen volume fraction (Fig. 1B-F). Intriguing, with the improvement of myocardial hypertrophy and fibrosis, the descending of LVEF and LVFS still appear in the same time with normal diet 5/6Nx mice. Therefore, retinol was elevated in CKD. Lowering serum retinol level by feeding vitamin A-free diet could attenuate myocardial hypertrophy and fibrosis but could not delay HFrEF progression in the late period of CKD.

### Establishing HFpEF after CKD mouse model by using 5/6Nx and retinol administration

**Figure 2:**
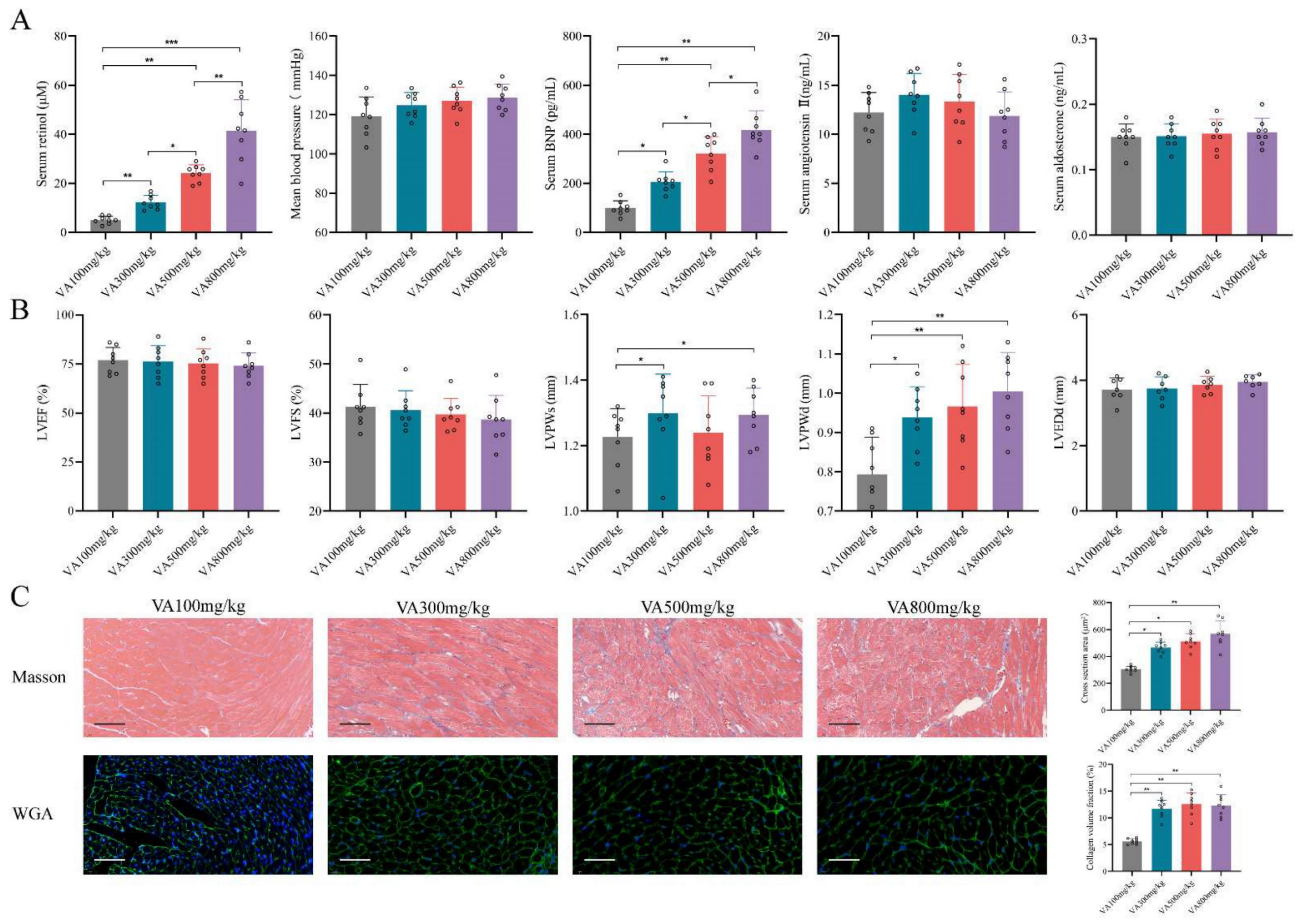
Retinol promotes myocardial hypertrophy and fibrosis in 5/6Nx. **(A)** Serum concentrations of retinol, BNP, angiotensin Ⅱ, aldosterone and mean blood pressure in 5/6Nx mice administrated vitamin A 100mg/kg, 300mg/kg, 500mg/kg or 800mg/kg for 6 weeks. **(B)** LVEF, LVFS, LVPWs, LVPWd, and LVEDd in 5/6Nx mice administrated vitamin A 100mg/kg, 300mg/kg, 500mg/kg or 800mg/kg for 6 weeks. **(C)** Masson staining and WGA staining in the cardiac ventricle of 5/6Nx mice administrated vitamin A 100mg/kg, 300mg/kg, 500mg/kg or 800mg/kg for 6 weeks. The right panels show collagen volume fraction calculated from Masson staining and cross section area calculated from WGA staining respectively. Data are shown as mean ± SD (*p < 0.05, **p < 0.01, ***p < 0.001).

**Figure 3:**
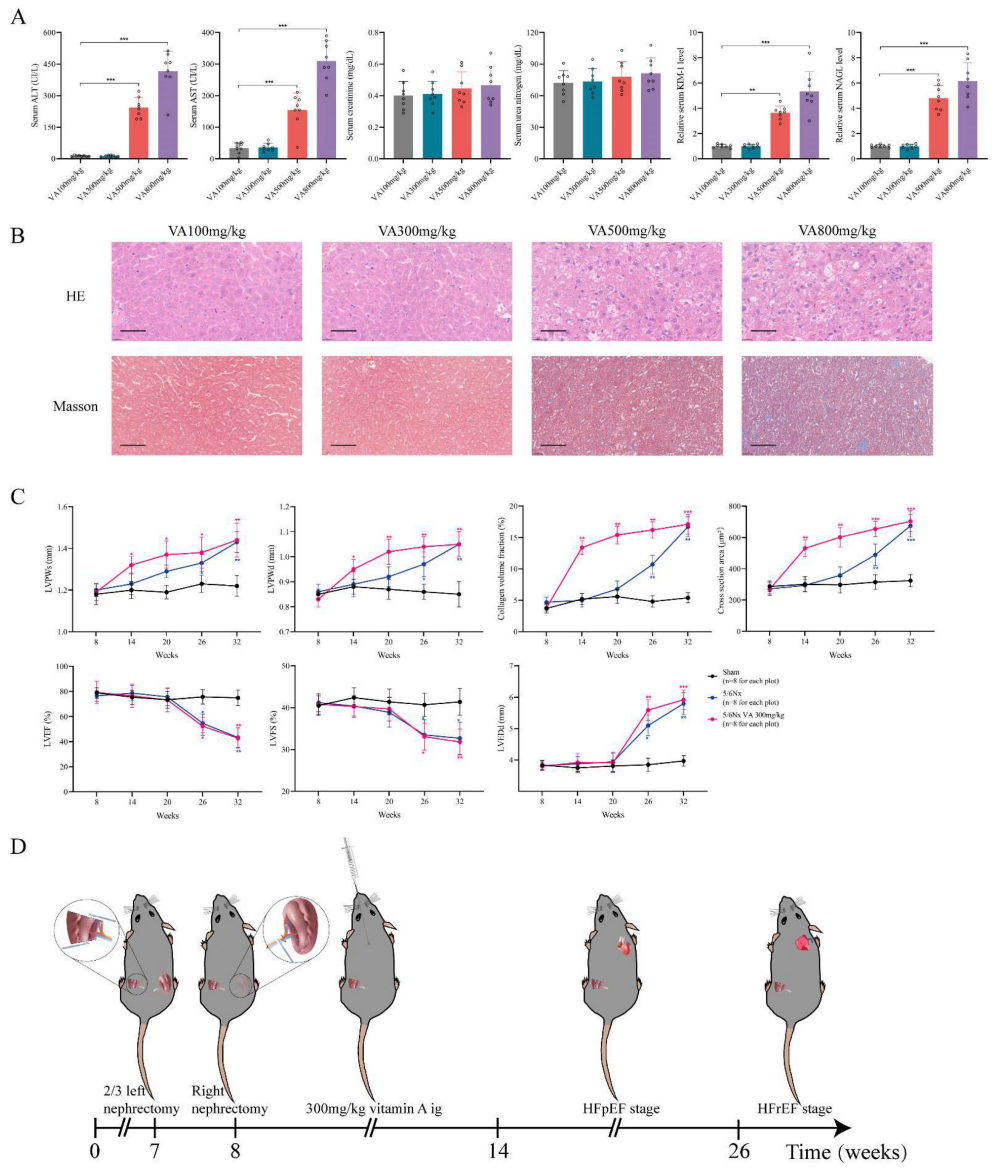
Establishment of HFpEF after CKD mouse model.** (A)** Serum concentrations of ALT, AST, creatinine, urea nitrogen, KIM-1 and NAGL in 5/6Nx mice administrated vitamin A 100mg/kg, 300mg/kg, 500mg/kg or 800mg/kg for 6 weeks. **(B)** HE and Masson staining of liver tissues from 5/6Nx mice administrated vitamin A 100mg/kg, 300mg/kg, 500mg/kg or 800mg/kg for 6 weeks. **(C)** LVPWs, LVPWd, collagen volume fraction, cross section area, LVEF, LVFS and LVEDd of 5/6Nx mice, 5/6Nx mice administrated vitamin A 300mg/kg and sham-operated mice from 8 weeks to 32 weeks. **(D)** Flow chart of establishing steps and the heart failure stages of HFpEF after CKD mouse model. Data are shown as mean ± SD (*p < 0.05, **p < 0.01, ***p < 0.001).

Since the retinol could promote myocardial hypertrophy and fibrosis but not HFrEF progression, we explored whether retinol induction could accelerate HFpEF in CKD mice before LVEF and LVFS declined. We conduct a vitamin A dosage gradient experiment by giving vitamin A 100mg/kg, 300mg/kg, 500mg/kg and 800mg/kg intragastrically every day for 6 weeks right after 5/6Nx. Observing after vitamin A administration, serum retinol level was climbing with the dosage of vitamin A increased, but blood pressure, aldosterone and angiotensin Ⅱ keep no significant difference (Fig. 2A). Significant myocardial fibrosis and hypertrophy with preserved LVEF and LVFS was exhibited by echocardiography and histochemical staining in vitamin A 300mg/kg, 500mg/kg and 800mg/kg, which met the definition of HFpEF (Fig. 2B, 2C). However, kidney injury and liver damage were exhibited during 5/6Nx mice receiving vitamin 500mg/kg and 800mg/kg (Fig. 3A, 3B). Therefore, we demonstrated vitamin A 300mg/kg administration could induce HFpEF in 5/6Nx CKD mice in 14 weeks without LVEF and LVFS decline as well as kidney injury and liver damage. During the establishment and observation of HFpEF after CKD mice model by 5/6 Nx and vitamin A 300mg/kg administration, 5.2% and 19.8% mice were died or failed to reach the HFpEF criteria for mice respectively. The overall success rate of HFpEF after CKD mice model is 75%.

Furthermore, we observed the 5/6 Nx mice receiving vitamin A 300mg/kg for 32 weeks to determine the progression of heart failure in CKD mice. Comparing to the 5/6 Nx mice, 5/6 Nx mice receiving vitamin A 300mg/kg demonstrated concentric cardiac hypertrophy, preserved ejection fraction and fibrosis in the early period of CKD. Finally, them would enter HFrEF stage in the same time in the late period of CKD (Fig. 3C, Fig. S1A and B). In conclusion, we established HFpEF after CKD mouse model by administrating vitamin A 300m/kg intragastrically every day for 6 weeks right after 5/6Nx. After 5/6Nx surgery at week 8, HFpEF after CKD mouse model exhibited HFpEF presentation from week 14 to week 26, and entered HFrEF stage from week 26 (Fig. 3D). Therefore week 14 to week 26 is the observation and operation window for HFpEF after CKD research.

### HFpEF after CKD mouse model exhibits diastolic dysfunction with preserved LVEF, cardiac hypertrophy, exercise intolerance and pulmonary edema

**Figure 4:**
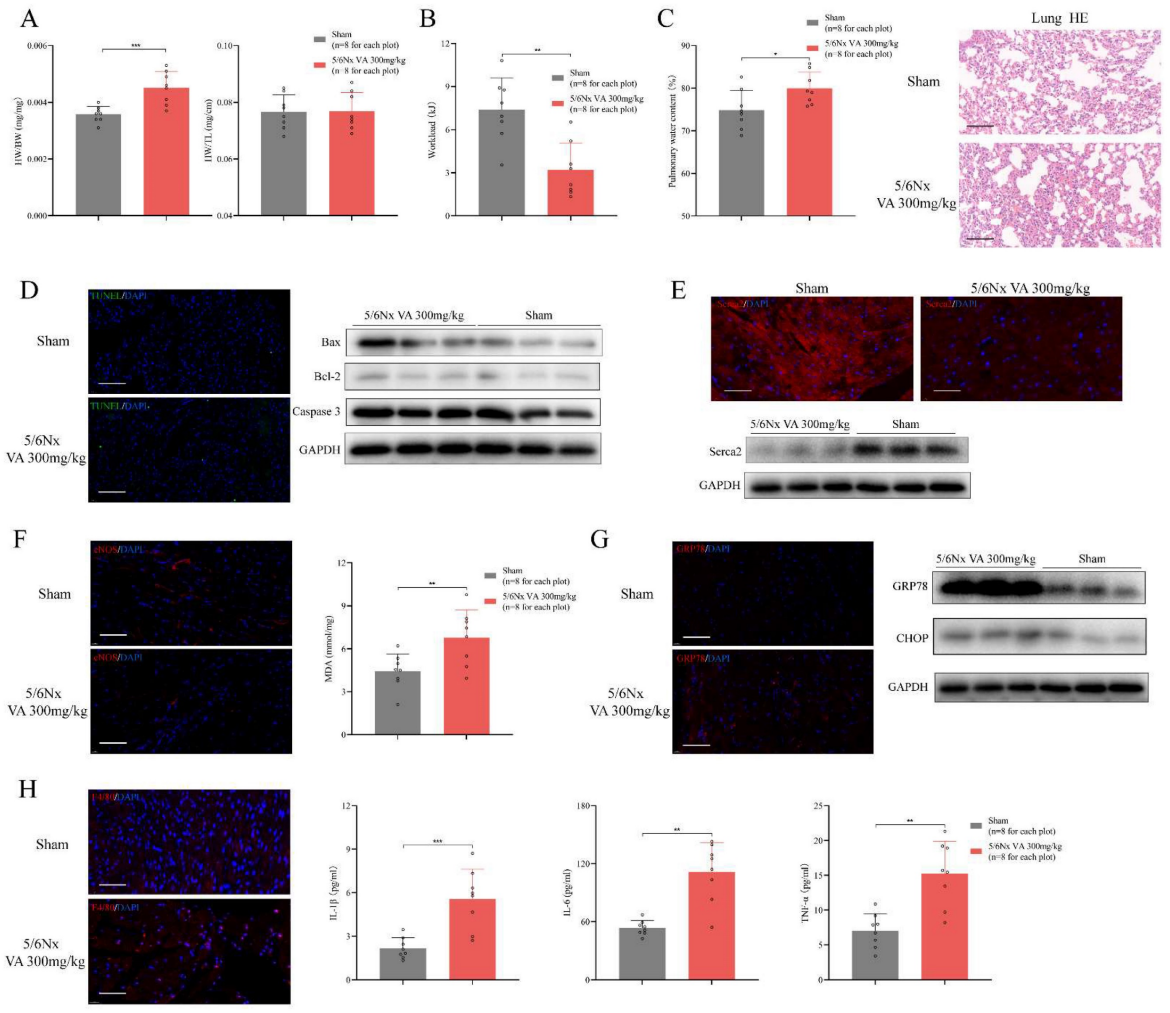
Major features of HFpEF after CKD mouse model. **(A)** Heart weight/body weight (HW/BW), heart weight/tibia length (HW/TL) of 5/6Nx mice administrated vitamin A 300mg/kg and sham-operated mice. **(B)** Workload of 5/6Nx mice administrated vitamin A 300mg/kg and sham-operated mice. **(C)** Pulmonary water content and HE staining of 5/6Nx mice administrated vitamin A 300mg/kg and sham-operated mice**. (D)** TUNEL (green) analysis and expressions of Bax, Bcl-2, Caspase 3 by western blotting in the cardiac ventricle of 5/6Nx mice administrated vitamin A 300mg/kg and sham-operated mice.** (E)** Immunofluorescence labelling of Serca2 (red) and expression of Serca2 by western blotting in the cardiac ventricle of 5/6Nx mice administrated vitamin A 300mg/kg and sham-operated mice.** (F)** Immunofluorescence labelling of eNOS (red) and MDA assay in the cardiac ventricle from 5/6Nx mice administrated vitamin A 300mg/kg and sham-operated mice. **(G)** Immunofluorescence labelling of GRP78 (red) and expressions of GRP78 and CHOP by western blotting in the cardiac ventricle of 5/6Nx mice administrated vitamin A 300mg/kg and sham-operated mice. **(H)** The left panel shows immunofluorescence labelling of F4/80 (red) in the cardiac ventricle of 5/6Nx mice administrated vitamin A 300mg/kg and sham-operated mice. Others show plasma concentrations of IL-1β, IL-6 and TNF-α in 5/6Nx mice administrated vitamin A 300mg/kg and sham-operated mice. Data are shown as mean ± SD (*p < 0.05, **p < 0.01, ***p < 0.001).

A qualified HFpEF mouse model should fulfil the major features including ejection fraction ≥50%, diastolic dysfunction, exercise intolerance, pulmonary edema and concentric cardiac hypertrophy 30. Following the HFpEF criteria, we evaluated these standards in the novel HFpEF after CKD mouse model. Heart mass, echocardiography and histochemical staining indicated evident diastolic dysfunction and concentric cardiac hypertrophy with preserved LVEF, LVFS and LVEDd in HFpEF after CKD mice during from 14 weeks to 26 weeks (Fig. 3C, Fig. 4A). Then, exercise testing was conducted to evaluated exercise capacity, in which HFpEF after CKD mice demonstrated severe exercise intolerance with a decline in exercise capacity to less than half of the controls (Fig. 4B). Pulmonary water content and lung Hematoxylin Eosin (HE) staining showed pulmonary edema in HFpEF after CKD mice (Fig. 4C). Therefore, the novel HFpEF after CKD mouse model basically meet the major features of HEpEF.

At the pathophysiological level, multiple processes including cardiomyocyte hypertrophy, interstitial fibrosis, apoptosis, impaired calcium handling may contribute to the progression of HFpEF 31. WGA and Masson staining indicated cardiomyocyte hypertrophy and interstitial fibrosis in HFpEF after CKD mice (Fig. 2C). TUNEL assay and western blotting showed apoptosis was slightly upregulated (Fig. 4D). While significant decrease of sarcoplasmic/endoplasmic reticulum Ca2+ ATPase 2 (Serca2) pointed impaired calcium handling in HFpEF after CKD mice (Fig. 4E). Downregulation of eNOS and lipid peroxidation were also observed using immunofluorescence histochemical staining and MDA Assay (Fig. 4F). Upregulated GRP78 and CHOP hinted endoplasmic reticulum stress was involved in HFpEF after CKD mice (Fig. 4G). And the infiltration of F4/80^+^ macrophage and the increase of serum IL-1β, IL-6 and TNF-α showed inflammatory state in HFpEF after CKD mice (Fig. 4H).

### HFpEF after CKD has distinct RNA expression features comparing to metabolic stress-induced HFpEF

To reveal the RNA profile in HFpEF after CKD, 6 HFpEF after CKD heart samples and 6 controls were subjected to next-generation RNA sequencing. 902 significantly upregulated RNA and 452 significantly downregulated RNA were detected in the heart tissues of HFpEF after CKD mice according to the following criteria: |log2 (fold change vs. control)| >1 and P<0.01 (Fig. S2A). Combining Go analysis and hierarchical networks of the abundance of GO terms using REVIGO, we predicted the enriched cellular processes in HFpEF after CKD were mainly clustered into four categories: inflammatory response including chemotaxis, cytokine binding, ion transport including potassium channel binding, cation channel activity, extracellular matrix including collagen trimer and others (Fig. S2B). Directed acyclic graph of GO terms also indicated inflammatory response and ion transport may be the key part in HFpEF after CKD (Fig. S2C). While NF-κb signal, PI3K/Akt signal and JAK/STAT signal may be the key pathways mediating HFpEF in CKD (Fig. S2D).

**Figure 5:**
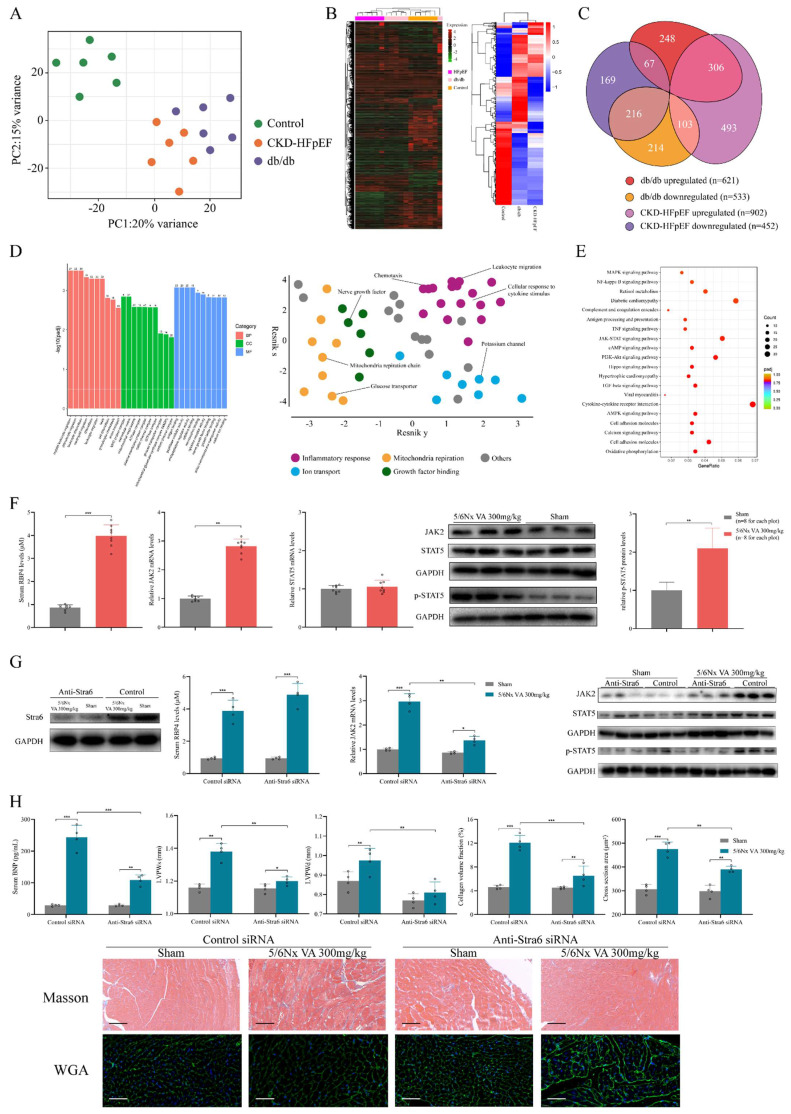
Transcriptomic differences between HFpEF after CKD and metabolic induced-HFpEF. RNAseq was performed on heart tissues from HFpEF after CKD mice (CKD-HFpEF, n=6), metabolic induced-HFpEF mice (db/db, n=6), and controls (n=6). **(A)**Principal component analysis for CKD-HFpEF mice, db/db mice and controls. **(B)** Hierarchical clustering analysis using all identified genes. **(C)** Venn diagram of DEGs for the 3 groups, their directions versus control, and relative portion unique or shared by CKD-HFpEF group and db/db group. **(D)** The left panel shows Go analysis of DEGs with different expression trends between CKD-HFpEF group and db/db group. The right panel shows hierarchical networks of the abundance of GO terms (Fisher's exact test, P < 0.05) using REVIGO. **(E)** KEGG analysis of DEGs with different expression trends between CKD-HFpEF group and db/db group. **(F)** Serum retinol level, the mRNA levels of JAK2 and STAT5 and expressions of JAK2, STAT5 and p-STAT5 in the cardiac ventricle of 5/6Nx mice administrated vitamin A 300mg/kg and sham-operated mice. **(G)** The cardiac expressions of Stra6, JAK2, STAT5, p-STAT5, serum RBP4 concentration and JAK2 mRNA level of Sham and 5/6Nx administrated vitamin A 300mg/kg mice after injection with control or anti-Stra6 siRNA. **(H)** Serum BNP, LVPWs, LVPWd, collagen volume fraction, cross section area, HE and Masson staining of Sham and 5/6Nx administrated vitamin A 300mg/kg mice after injection with control or anti-Stra6 siRNA. Data are shown as mean ± SD (*p < 0.05, **p < 0.01, ***p < 0.001).

Also, we compared RNA profile of HFpEF after CKD mice to the db/db HFpEF mice, in order to explore the RNA heterogeneity between HFpEF after CKD and metabolic induced-HFpEF. Principal component analysis (PCA) showed HFpEF after CKD group, db/db group and control group were separated clearly. Although samples from HFpEF after CKD group and db/db group were close to each other, there was no overlap between these two groups (Fig. 5A). Unsupervised hierarchical cluster analysis also identified 3 distinct groupings, with 100% of HFpEF after CKD, 83% of HFrEF, and 83% of controls clustering within their category (Fig. 5B). Taking together, these indicated HFpEF after CKD mice had distinct RNA pattern comparing to metabolic stress-induced HFpEF. For differentially expressed genes (DEGs) from HFpEF after CKD group and db/db group, nearly half were shared similar expression pattern, whereas about 11% went in opposite directions (Fig. 5C). To explain which processes the differences mainly lie, DEGs with different expression trends were subjected to GO and KEGG analysis. Go and REVIGO hierarchical network indicated the differences between HFpEF after CKD and metabolic stress-induced HFpEF mainly presented in inflammatory response, ion transport, mitochondria respiration and growth factor binding (Fig. 5D). And retinol metabolism, JAK/STAT signal, diabetic cardiomyopathy, TGF-β signal and Hippo signal were mainly different pathways (Fig. 5E).

### Retinol activates JAK2/STAT5 signals in HFpEF after CKD

To explore the potential mechanism that retinol induces HFpEF in CKD, we reviewed on the metabolism of retinol. Circulating retinol is carried by retinol binding protein 4 (RBP4). After reaching the target cell, retinol was released from RBP4 and the unloaded RBP4 can stimulate Toll-like receptor 4 and activation c-Fos/c-Jun pathway. After uptake by Stra6, retinol is converted into retinoic acid intracellularly, which activates retinoic acid receptor (RAR) and retinoid X receptor (RXR). These receptors form heterodimers and activate JAK/STAT pathway by upregulating JAK2 and phosphorylating STAT5 32. Also, KEGG analysis of RNA-seq of HFpEF after CKD heart tissues predicted high enrichment in JAK/STAT pathway (Fig. S2D). And the RNA sequencing detected significantly increase of JAK2, RAR, RXR and RBP4 mRNA (Fig. S2A). Combining our bioinformation analysis of RNA-seq with the literature review of retinol metabolism, we focused on the retinol activated JAK/STAT pathway. Although JAK/STAT family has many subtypes and branch paths, most of them are activated by cytokines such as interferon and IL-2 family 33. While JAK2 and STAT5 are the main target by retinol embolism 34. RT-qPCR validated the upregulated expression of JAK2 and RBP4, while STAT5 expression had no significant change between HFpEF after CKD and controls. Then, we discovered phosphorylated STAT5 (p-STAT5) was increased along with JAK2 by western blotting (Fig. 5F). In order to determine whether the increase of JAK-2 and p-STAT5 are directly mediated by retinol, we used Stra6 siRNA to block retinol uptake. With the decrease of Stra6, expression of RBP4, JAK2 and p-STAT5 were also reduced significantly (Fig. 5G). The BNP level, cardiac hypertrophy and interstitial fibrosis were also improved after administrated Stra6 siRNA in HFpEF after CKD mice (Fig. 5H).

### Inhibiting STAT5 phosphorylation attenuates HFpEF progression in CKD

**Figure 6:**
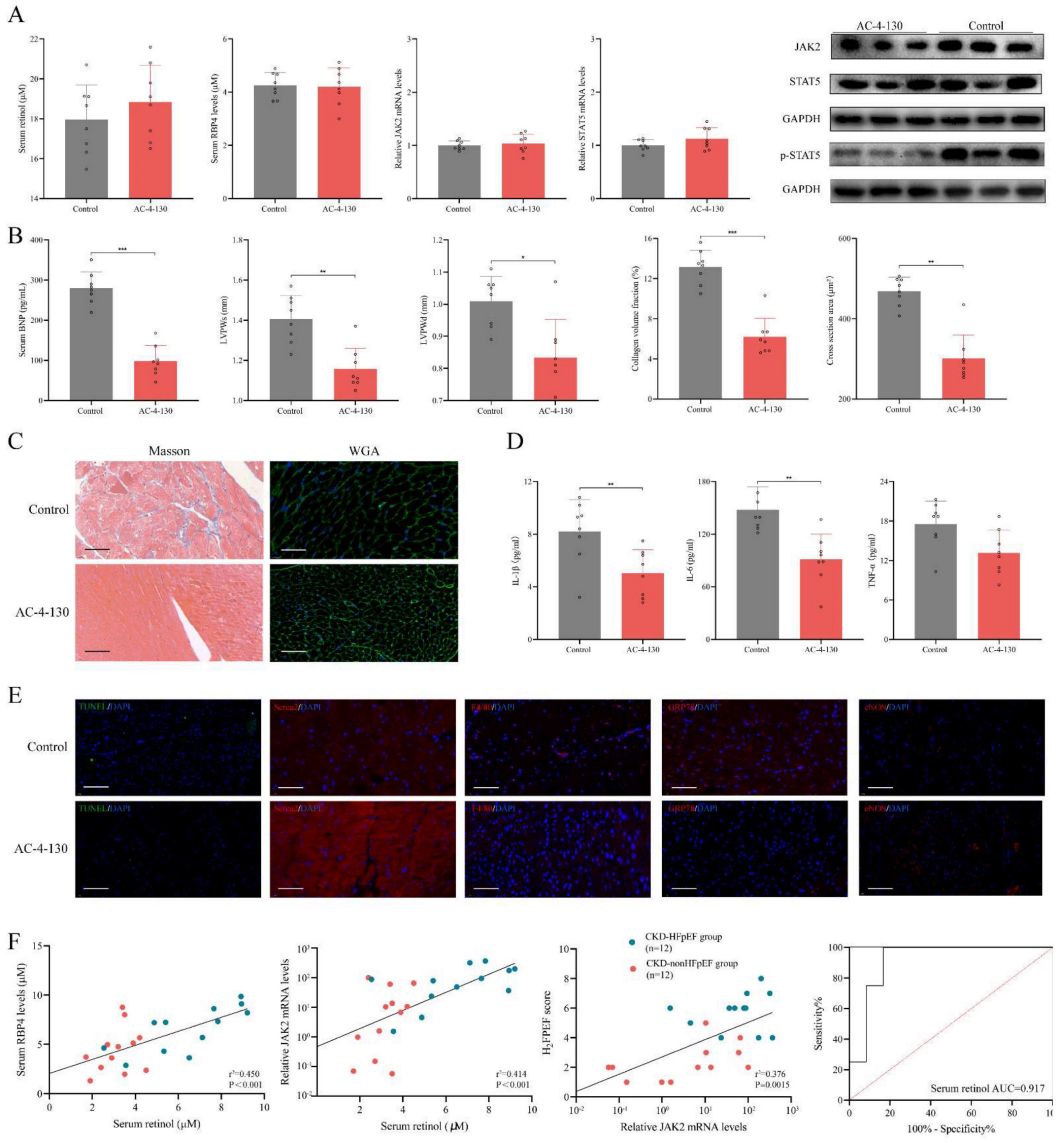
Inhibiting STAT5 phosphorylation suppresses HFpEF progression in CKD. **(A)** Serum concentration of retinol, RBP4 and relative mRNA levels of JAK2, STAT5 and expressions of JAK2, STAT5 and p-STAT5 in the cardiac ventricle of 5/6Nx mice administrated vitamin A 300mg/kg after injection with AC-4-130 or control. **(B)** Serum BNP, LVPWs, LVPWd, collagen volume fraction and cross section area of 5/6Nx mice administrated vitamin A 300mg/kg after injection with AC-4-130 or control.** (C)** Masson and WGA staining of the cardiac ventricle of 5/6Nx mice administrated vitamin A 300mg/kg after injection with AC-4-130 or control.** (D)** Plasma concentrations of IL-1β, IL-6 and TNF-α in 5/6Nx mice administrated vitamin A 300mg/kg after injection with AC-4-130 or control.** (E)** TUNEL (green) analysis and immunofluorescence labelling of Serca2 (red), F4/80 (red), GRP78 (red), eNOS (red) in the cardiac ventricle of 5/6Nx mice administrated vitamin A 300mg/kg after injection with AC-4-130 or control.** (F)** Relationship serum retinol concentration to serum RBP4 concentration, blood relative JAK2 mRNA level in CKD patients. The relationship between blood relative JAK2 mRNA level and H_2_FPEF score in CKD patients. ROC curves of serum retinol for prediction HFpEF in CKD patients. AUC indicates the area under the curve. Data are shown as mean ± SD (*p < 0.05, **p < 0.01, ***p < 0.001).

We used STAT5 phosphorylation small molecule inhibitor AC-4-130 to explore the role p-STAT5 mediated by retinol plays in the HFpEF progression in CKD 35. After injected AC-4-130, p-STAT5 was significantly decreased while serum retinol level and RBP4, JAK2 and STAT5 mRNA remained calm (Fig. 6A). There is no significant difference between CKD group and CKD treated with AC-4-130 group in serum creatinine, serum urea nitrogen, serum angiotensin Ⅱ and serum aldosterone. HE staining and Masson staining also showed no evident change after CKD treated with AC-4-130 (Fig. S3). Therefore, STAT5 inhibitor AC-4-130 may not attenuate CKD progression. However, the serum BNP level and cardiac hypertrophy evaluated by LVPWs and LVPWd were tremendously improved (Fig. 6B). Masson and WGA staining also showed myocardiocyte and interstitial fibrosis were restrained by AC-4-130 (Fig. 6C). Furthermore, impaired calcium handling evaluated and endoplasmic reticulum stress were attenuated. But the levels of apoptosis, endoplasmic reticulum stress and eNOS had no significant change between HFpEF after CKD administrated AC-4-130 group and controls (Fig. 6E). Macrophage infiltration and IL-1β, IL-6, TNF-α climbing in HFpEF after CKD were alleviated by STAT5 phosphorylation inhibitor AC-4-130, too (Fig. 6D, 6E). Therefore, JAK2/STAT5 plays great part in HFpEF progression in CKD. Inhibiting STAT5 phosphorylation may be the potential target to intervene HFpEF after CKD.

In CKD patients' blood samples, we found serum RBP4 level and blood JAK2 mRNA level were closely relevant to serum retinol level. And blood JAK2 mRNA level was positively correlated with HF_2_PEF clinical score. Since the strong link between serum retinol and HFpEF after CKD, we explored the prediction value of serum retinol for HFpEF occurrence in CKD patients. We discovered serum retinol could predict the HFpEF occurrence in CKD patients with AUC 0.917 [0.789-1.000] (Fig. 6F). Based on receiver operating characteristic analysis and the Youden index, the cutoff value for serum retinol was established as 4.690µM, which indicated that CKD patients with serum retinol level >4.690µM may have great risk for HFpEF.

## Discussion

It has long been recognized that renal dysfunction and heart failure were a detrimental combination 36. While chronic kidney disease seems to prefer HFpEF than HFrEF as an accompanying comorbidity. Studies have demonstrated that chronic kidney disease was significantly related to new-onset HFpEF, but was not a predictor for the onset of HFrEF, which greatly increased cardiovascular mortality and all-cause mortality of CKD patients 37, 38. For this specific subtype of HFpEF, lack of practical animal model makes it hard to peek the unique pathogenesis and to discover targeted intervention for HFpEF after CKD.

In this study, we determined the heart failure characteristics and dynamic progression in CKD mice. Based on these features, we established the novel HFpEF after CKD mouse model by 5/6 subtotal nephrectomy and retinol administration. To verify the typicality and representativeness of our model, we evaluated the 5 major features of HFpEF: diastolic dysfunction, ejection fraction ≥50%, concentric cardiac hypertrophy, exercise intolerance and pulmonary edema macroscopically. We also observed cardiomyocyte hypertrophy, interstitial fibrosis, active apoptosis, impaired calcium handling, an imbalance between eNOS and oxidative stress, engaged endoplasmic reticulum stress and evident macrophage infiltration in HFpEF after CKD mouse model microscopically. RNSseq revealed distinct gene expression patterns between HFpEF after CKD and metabolic induced-HFpEF mainly manifested in inflammatory response, ion transport, mitochondria respiration and growth factor binding. JAK2/STAT5 signal activated by retinol may be the key pathways prompts cardiac hypertrophy, interstitial fibrosis and HFpEF progression behind the scenes. And targeting STAT5 phosphorylation may be a potential way to intervene HFpEF after CKD.

To our knowledge, this study is the first to establish the mouse model of HFpEF after CKD. At present, the animal models of HFpEF can be divided into these following categories according to the accompanying comorbidities: aging induced HFpEF, hypertension induced HFpEF, obesity and diabetes induced HFpEF, atrial fibrillation induced HFpEF, pulmonary hypertension induced HFpEF, renal insufficiency induced HFpEF and multi-comorbidities induced HFpEF model. Among these models, only few could reach the criteria of HFpEF and remain stable in observation time. AIU mouse model (classified as hypertension induced HFpEF), db/db mouse model and ZSF1 rat model (classified as obesity and diabetes induced HFpEF) and multi-hit mouse model (classified as multi-comorbidities induced HFpEF model) are representative HFpEF models considering the stability and manifestation 14, 16, 30, 39-42. Although HFpEF after CKD need two stages surgery of 5/6 subtotal nephrectomy, our model fulfils the criteria of HFpEF for mice and remain stable in relatively long time. Compared to these common HFpEF animal models, our HFpEF mouse also has several features: accompany with chronic kidney disease, prominent myocardial hypertrophy, interstitial fibrosis and mild apoptosis. The key induction of our HFpEF after CKD mouse model is retinol. Retinol is the main circulating forms and key metabolite of vitamin A which is available into the human diet by intake of either food containing preformed vitamin A or carotenoids. Retinoids are vital for human health and play a crucial role in the regulation of nocturnal vision, reproduction, immune function, and cell differentiation 43. Kidney plays an important role in vitamin A homeostasis. Approximately 50% of the circulating retinol pool originates in the kidneys, while more than 99% of retinol is reabsorbed by the proximal renal tubule 44. Chronic kidney disease patients have been reported to have high circulating levels of retinol, possibly due to a combination of decreased retinol clearance and reduced conversion of retinol 45. As the carrier of retinol in circulation RBP4 has been confirmed by many studies to be positively correlated with various cardiovascular diseases including heart failure 46. But the role of retinol in heart failure was neglected until the newly reported research focusing on the pro-heart failure effect of retinol in CKD. This study found CKD induced the expression of GPR68 in circulating monocytes via altered CLOCK activation by increasing serum levels of retinol and its binding protein (RBP4). The high-GPR68-expressing monocytes increased inflammatory cytokines and cardiac infiltration, which exacerbated inflammation and fibrosis of heart 28. Based on the clinical features of CKD patients and retinol pro-heart failure effect, we used retinol to induce HFpEF after CKD mouse model.

Our study has several limitations. First, the stability of HFpEF after CKD mouse model need to be improved because HFpEF would switch to HFrEF in the late period of CKD. Though it is rare in clinical, progression to HFrEF or others within a variable amount of time is a common problem for many HFpEF animal model including Fischer 344 rat (evolving into eccentric hypertrophy), Dahl salt-sensitive rat (evolving into HFrEF), db/db mouse (evolving into diabetic cardiomyopathy) 47, 48. It suggests that in these models, HFpEF is merely a temporary step to the development to HFrEF and the specific comorbidity combination causes the most detrimental progression of HFpEF is unknown. Second, the evaluations of diastolic dysfunction are indirect. Differences between rodents and human in heart rates and size limit diastolic function measurements, including both noninvasive techniques and invasive hemodynamics. Third, the experiments of retinol activating JAK2/STAT5 signals to prompt HFpEF in CKD is preliminary. But according to our histochemical staining of HFpEF after CKD mice treated with AC-4-13, JAK2/STAT5 activation may induce myocardial hypertrophy and fibrosis by affecting calcium handling, endoplasmic reticulum stress, macrophage infiltration or cytokine surge (Fig. 6C-E). Moreover, among the multiple pathways that retinol is able to activate including RAR/RXR signals, TLR signals, MAPK signals, we only investigated JAK/STAT signals according to our RNA-seq. And we could not rule out the interference of other retinol activated pathways. Therefore, we hope to illustrate the functions of other retinol activated signals in HFpEF after CKD in further investigations.

In conclusion, this study offers a novel method to establish the HFpEF after CKD mouse model and the first analysis of the differences of gene expression patterns between HFpEF after CKD and metabolic induced-HFpEF. An underlying mechanism was revealed. Serum accumulation of retinol in CKD prompts myocardial hypertrophy and fibrosis by activating JAK2/STAT5. And the STAT5 phosphorylation inhibitor may be a potential intervention target for HFpEF after CKD.

## Supplementary Material

Supplementary figures and table.

## Figures and Tables

**Figure 7:**
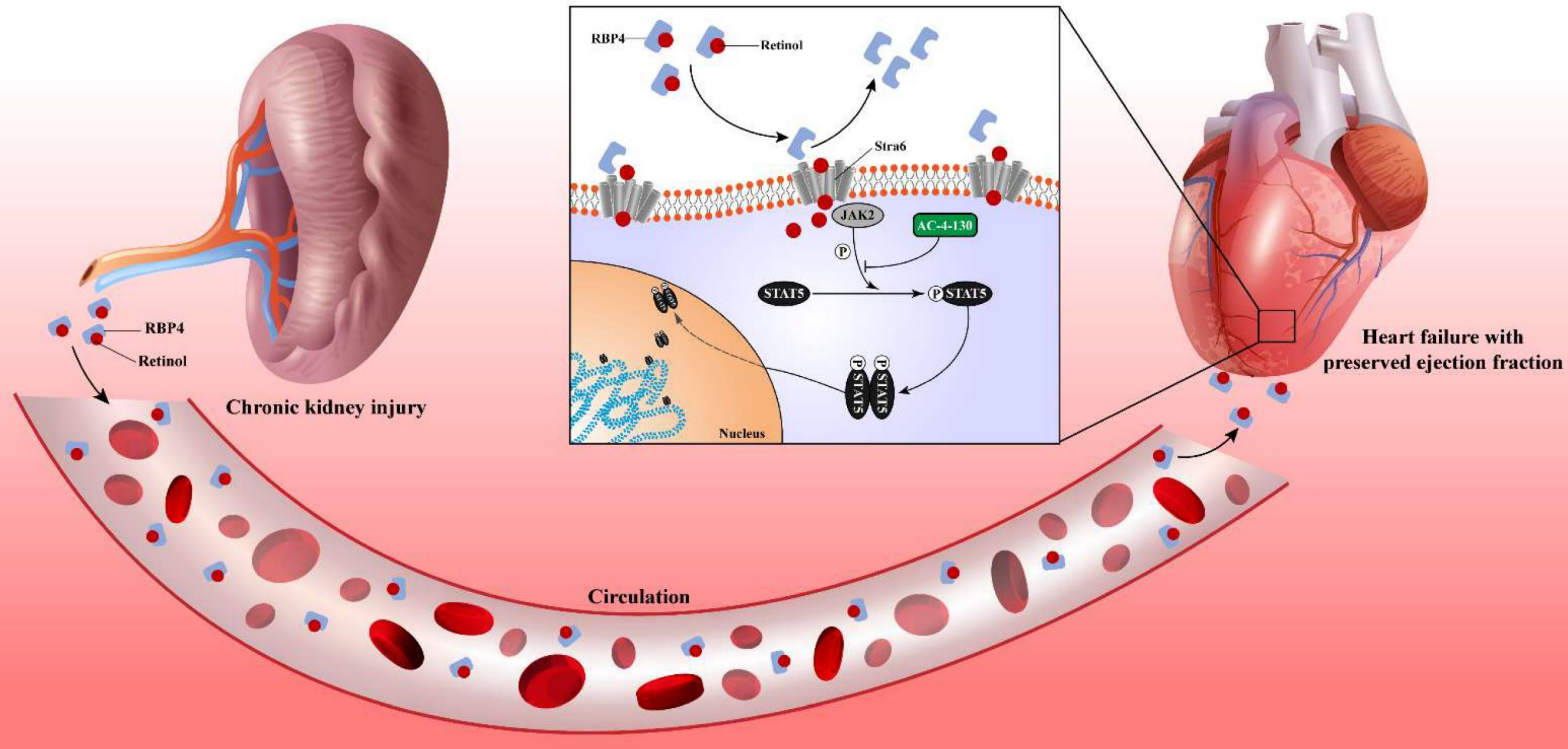
Chronic kidney disease causes serum retinol level rises. Elevated Retinol prompts cardiac hypertrophy and fibrosis through activating JAK2 and phosphorylating STAT5, which may cause the progression of heart failure with preserved ejection fraction progression in chronic kidney disease.
