# Supplementary material for: A novel mouse model of heart failure with preserved ejection fraction after chronic kidney disease induced by retinol through JAK/STAT pathway
Source: Int J Biol Sci. 2023 Jul 16;19(12):3661–77. doi: 10.7150/ijbs.83432 (PMC10411473; doi:10.7150/ijbs.83432)
Supplement: Supplementary file 1 — Supplementary figures and table. [file ijbsv19p3661s1.pdf]

Table S1: Baseline demographic and clinical characteristics of CKD-HFpEF, CKD-nonHFpEF and control groups

Variable	Controls (n=12)	CKD-HFpEF (n=12)	CKD-nonHFpEF (n=12)	P value
Age, y	60.4± 2.2	60.0±2.8	60.2±2.0	0.91
Female	9 (75%)	9 (75%)	9 (75%)	> 0.99
Medications				
ACEi or ARB	2 (16.7%)	7 (58.3%)	5 (41.7%)	0.11
ARNI	0 (0%)	3 (25%)	0 (0%)	0.038
β-Blocker	3 (25%)	6 (50%)	7 (58.3%)	0.23
SGLT2i	0 (0%)	8 (66.7%)**, †	2 (16.7%)	<0.001
Loop diuretic	0 (0%)	6 (50%)*	5 (41.7%)*	0.017
Mineralocorticoid RA	1 (8.3%)	4 (33.3%)	3 (25%)	0.32
Comorbidities				
Hypertension	4 (33.3%)	11 (91.7%)**	8 (66.7%)	0.012
Type2 Diabetes Mellitus	2 (16.7%)	10 (83.3%)**, †	4 (33.3%)	0.003
Coronary artery disease	2 (16.7%)	3 (25%)	2 (16.7%)	0.84
Atrial fibrillation or flutter	0 (0%)	1 (8.3%)	0 (0%)	0.36
COPD	1 (8.3%)	3 (25%)	1 (8.3%)	0.39
Sleep Apnea	0 (0%)	1 (8.3%)	1 (8.3%)	0.59
Dialysis	0 (0%)	5 (41.7%)*	4 (33.3%)	0.044
BMI, kg/m^2	20.8±2.4	22.6±2.3	21.4±1.6	0.13
Laboratory Examination				
eGFR, mL/min/1.73 m^2	81.3±4.8	41.5±9.0***	45.4±10.8***	<0.001
HbA1c, %	6.9±0.34	6.2±0.56*	6.8±0.33	0.011
Echocardiography				
LVEF, %	62.9±3.7	62.2±4.4	64.4±4.1	0.92
LVEDD, cm	4.1±0.30	4.2±0.29	4.2±0.24	0.91
LV posterior wall, mm	94.8±4.6	106.3±5.6***, ††	97.0±4.5	<0.001
LVMI, g/m^2	91.3±5.8	108.7±10.2***, †††	87.1±7.6	<0.001

Continuous variables are presented as are median ± standard deviation. Categorical variables are reported as n%. P value displayed for one-way ANOVA was used for continuous variables with differences between Gaussian distribution. Continuous variables under non-normally distribution were analyzed by Kruskal–Wallis test. P value for categorical variables were analysed by either the chi-square test or Fisher's exact test. Post hoc between-group comparison statistics (Wilcoxon)

*are as follows: *P < 0.05 vs control, **P ⩽0.01 vs control, ***P≤0.001 vs control; † P < 0.05 vs CKD-nonHFrEF, ††P < 0.01 vs CKD-nonHFrEF, †††P < 0.001 vs CKD-nonHFrEF. CKD-HFpEF indicates heart failure with preserved ejection fraction in chronic kidney disease patients; CKD-nonHFpEF, chronic kidney disease patients without heart failure with preserved ejection fraction; ACEi, angiotensin-converting enzyme inhibitor; ARB, angiotensin II receptor blocker; ARNI, angiotensin receptor II blocker - neprilysin inhibitor; β-Blocker, β-receptor antagonist; SGLT2i, sodium-dependent glucose transporters 2 inhibitor; Mineralocorticoid RA, mineralocorticoid receptor antagonist; COPD, chronic obstructive pulmonary disease; BMI, body mass index; eGFR, estimated glomerular filtration rate; HbA1c, glycosylated hemoglobin, type A1C; LVEF, left ventricular ejection fraction; LVEDD, left ventricular end-diastolic diameter; LV posterior wall, left ventricular posterior wall; and LVMI, left ventricular mass index.*

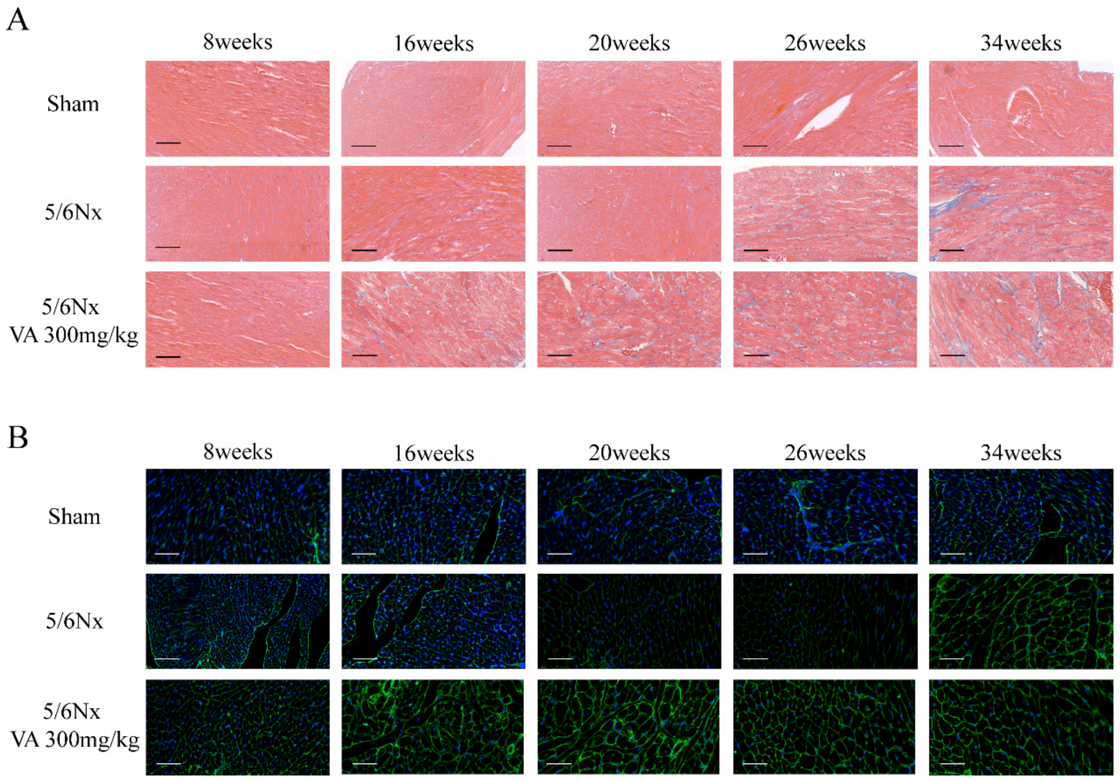

Figure S1 *Masson and WGA staining of the cardiac ventricle of 5/6Nx mice, 5/6Nx mice administrated vitamin A 300mg/kg and sham-operated mice from 8 weeks to 32 weeks.* ***(A)*** *Masson staining of the cardiac ventricle of 5/6Nx mice, 5/6Nx mice administrated vitamin A 300mg/kg and sham-operated mice from 8 weeks to 32 weeks.* ***(B)*** *WGA staining of the cardiac ventricle of 5/6Nx mice, 5/6Nx mice administrated vitamin A 300mg/kg and sham-operated mice from 8 weeks to 32 weeks.*

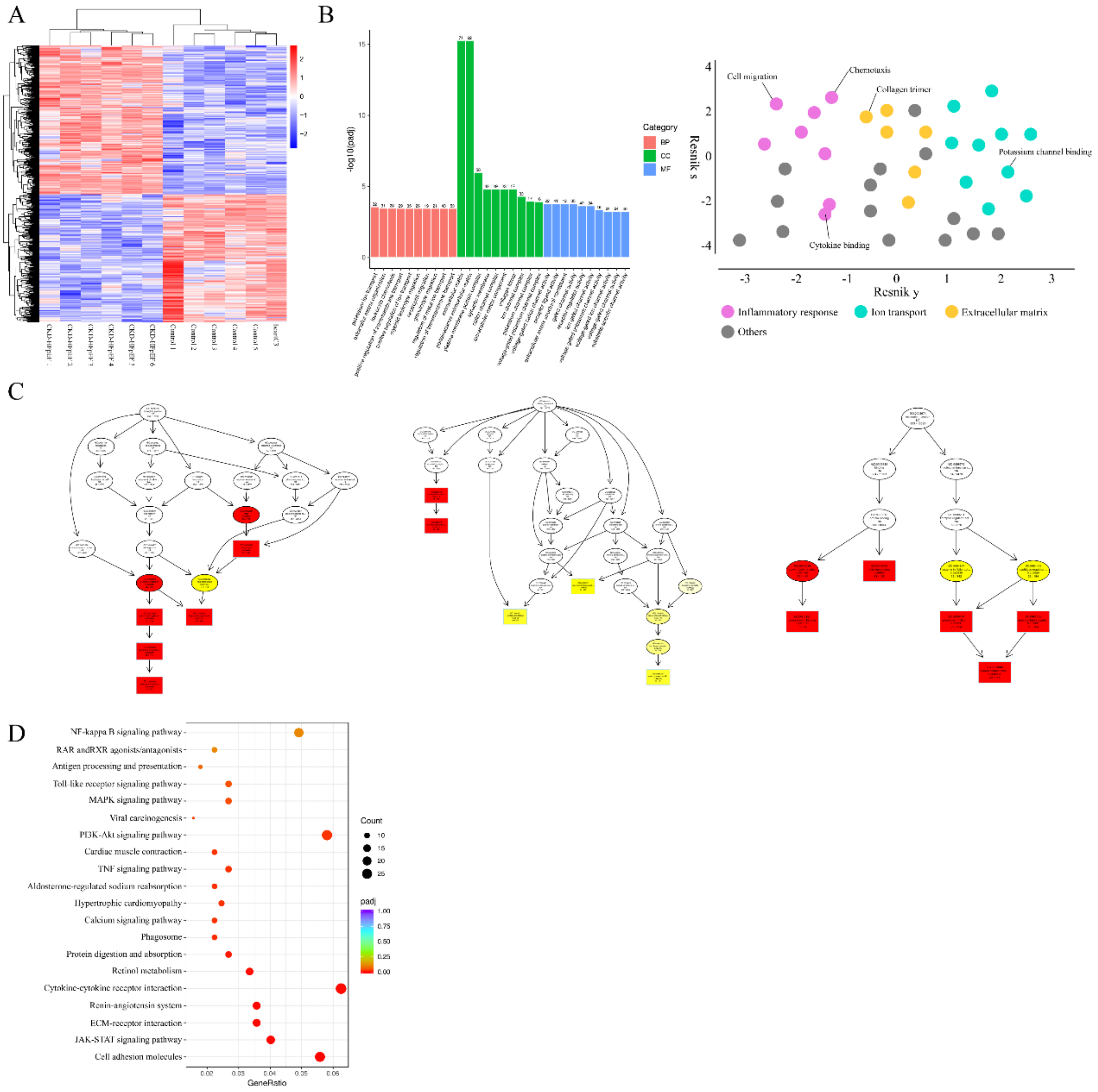

Figure S2 *RNA profile of HFpEF after CKD mice.* ***(A)*** *Heatmap of DEGs in the heart tissues of HFpEF after CKD (CKD-HFpEF) mice.* ***(B)*** *Go analysis of DEGs in the heart tissues of HFpEF after CKD mice and hierarchical networks of the abundance of GO terms (Fisher's exact test, P < 0.05) using REVIGO.* ***(C)*** *Directed acyclic graph of GO terms of the heart tissues from HFpEF after CKD (CKD-HFpEF) mice.* ***(D)*** *KEGG analysis of DEGs in the heart tissues of HFpEF after CKD mice*

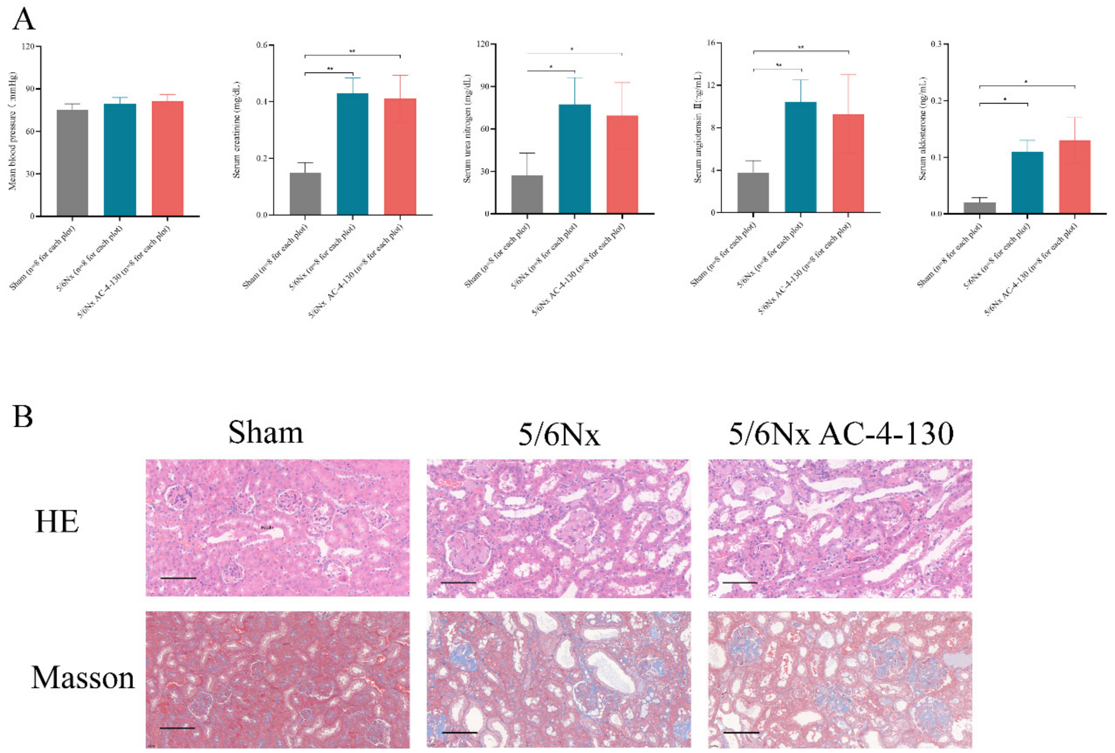

Figure S3 *Effect of AC-4-130 on CKD mice.* ***(A)*** *Blood pressure and serum concentrations of creatinine, urea nitrogen, angiotensin II and aldosterone in Sham, 5/6Nx mice and 5/6Nx mice injected with AC-4-130.* ***(B)*** *HE and Masson staining of kidney tissue of Sham, 5/6Nx mice and 5/6Nx mice injected with AC-4-130. Data are shown as mean ± SD (*p < 0.05, **p < 0.01, ***p < 0.001).*
